# Noggin inactivation affects the number and differentiation potential of muscle progenitor cells *in vivo*

**DOI:** 10.1038/srep31949

**Published:** 2016-08-30

**Authors:** Domiziana Costamagna, Hendrik Mommaerts, Maurilio Sampaolesi, Przemko Tylzanowski

**Affiliations:** 1 Translational Cardiomyology Lab, Stem Cell Biology and Embryology, Dept. Development and Regeneration, KU Leuven, Belgium; 2 Laboratory of Experimental Medicine and Clinical Pathology, Dept. Clinical and Biological Sciences, University of Turin, Italy; 3 Department of Development and Regeneration, Laboratory for Developmental and Stem Cell Biology, Skeletal Biology and Engineering Research Centre, KU Leuven, Belgium; 4 Division of Human Anatomy, Dept. of Public Health, Experimental and Forensic Medicine, University of Pavia, Italy; 5 Department of Biochemistry and Molecular Biology, Medical University, Lublin, Poland

## Abstract

Inactivation of Noggin, a secreted antagonist of Bone Morphogenetic Proteins (BMPs), in mice leads, among others, to severe malformations of the appendicular skeleton and defective skeletal muscle fibers. To determine the molecular basis of the phenotype, we carried out a histomorphological and molecular analysis of developing muscles *Noggin*^−/−^ mice. We show that in 18.5 dpc embryos there is a marked reduction in muscle fiber size and a failure of nuclei migration towards the cell membrane. Molecularly, the absence of Noggin results in an increased BMP signaling in muscle tissue as shown by the increase in SMAD1/5/8 phosphorylation, concomitant with the induction of BMP target genes such as *Id1, 2, 3* as well as *Msx1*. Finally, upon removal of *Noggin*, the number of mesenchymal Pax7^+^ muscle precursor cells is reduced and they are more prone to differentiate into adipocytes *in vitro*. Thus, our results highlight the importance of Noggin/BMP balance for myogenic commitment of early fetal progenitor cells.

Noggin is a glycosylated, secreted 64 kDa protein homodimer and one of the many extracellular antagonists of the Bone Morphogenetic Protein (BMP) signaling pathway. Similar to *Chordin*, *Chordin-like*, *Follistatin*, *Fsrp* and *Cerberus*, *Noggin* encodes a protein that directly binds to the BMP (BMP2, BMP4, BMP5, BMP7) or Growth Differentiation Factor (GDF5, GDF6) ligands with various affinities^1^,^2^. By preventing interaction of the ligands with their receptor, Noggin interferes with the downstream activation of the signaling cascade.

Loss of Noggin in mice resulted in an embryonic lethal phenotype that is characterized by different developmental defects such as exencephaly, axial outgrowth defects with a loss of the caudal vertebrae, failure of neural tube closure, excessive cartilage formation and fused joints to mention a few^3^,^4^.

During embryonic development, the bilaterally organized somites differentiate into a ventral-medial part, called the sclerotome, and a dorsal-lateral part, called the dermomyotome. The sclerotome gives rise to the cartilage and the bone of the vertebral column, while the dermomyotome develops into muscle, endothelia, cartilage, connective tissue and dermis.

At 9.5 dpc, delamination and migration of Pax3 positive (Pax3^+^) cells originating from the dermomyotome enables the differentiation of muscle progenitor cells in the myotome and in the limb. At 10.5–12.5 dpc, the first wave of myogenesis (embryonic myogenesis) takes place. Embryonic myoblasts fuse with each other and differentiate into large primary myofibers^5^. As most of the myoblasts remain in a committed and undifferentiated state, the number of myofibers produced in this first wave is limited. These primary fibers serve to form the basic muscle pattern^6^. Another cell type, which is Pax7^+^, remains undifferentiated. These cells are present from early stages onwards and give rise to fetal myoblasts^7^. Their proliferation is triggered by mitogens secreted by the primary fibers and they will differentiate into many smaller secondary myofibers during the secondary wave of myogenesis (14.5–16.5 dpc), or fetal myogenesis^8^.

Yet not all Pax7^+^ cells proliferate and differentiate but some remain in an undifferentiated state and become activated in the postnatal life following triggers like trauma or physical exercise. These cells are called satellite cells when they can be morphologically identified as mononucleated cells residing between the myofiber plasma membrane and the basal lamina (from 17.5 dpc onwards). They are considered to form the stem cell niche responsible for the growth and restoration of the muscle^9^.

The dermomyotomal and sclerotomal somitic populations are subject to the intricate crosstalk of several signaling cascades including WNT, Sonic hedgehog (SHH), and Bone Morphogenetic proteins (BMPs), ensuring a regulated differentiation of these lineages. WNT signaling from the overlying epidermis and the roof plate of the neural tube induces the expression of dermomyotome specific genes, while SHH signaling from the notochord and the floor plate of the neural tube induces sclerotomal gene expression^10^. In addition, BMP expression in the epidermis, the roof plate of the neural tube and the lateral plate mesoderm prevent the differentiation of myogenic lineage^11^. Differently, Noggin, present in the roof and floor plate of the neural tube, blocks this BMP action and therefore allows for the myogenic precursors to differentiate^12^,^13^. This balance between multiple signaling pathways results, among others, in the restricted expression of myogenic regulatory factors (MRF) and *Pax* genes in myogenic cell populations^14^.

Besides its role during the patterning of the somite, BMP signaling also affects the differentiation of myofibers. The effect of BMP signaling was shown to depend on the developmental stage and the progression along the myogenic program. Whereas the differentiation of embryonic myoblasts was shown to be insensitive to BMP signals, the fetal myoblasts and the Pax7^+^ precursors require a decrease of the BMP signaling in order to allow further myogenic differentiation^8^.

We have reported before that the *Noggin* null *(Noggin*^−/−^) mice presented a muscle defect during the final stages of in utero development^4^. From early stages onwards the size of the muscles was reduced. At 18.5 dpc, the *Noggin*^−/−^ embryos displayed a dramatic muscle phenotype characterized by disorganized myofibers that failed to align. In addition, the number of multinucleated myofibers was reduced, more mononucleated myoblasts were found and the nuclei failed to migrate towards the plasma membrane in the *Noggin*^−/−^ muscles. The defects appear late during the development since we could not detect them at 16.5 dpc, indicating that the phenotype was induced between 16.5 dpc and 18.5 dpc (fetal myogenesis). While we made that observation, the molecular basis of that phenotype was not investigated. Here we focused on the molecular mechanisms underlying the phenotype. Our results suggest that the absence of Noggin, and consequently BMP hypersignaling, leads to the decrease of the number of muscle progenitor cells and their phenotypic shift towards adipocytes *in vitro*.

## Results

### *Noggin* ^−/−^ muscle fibers display a reduced fiber thickness

**Figure 1:**
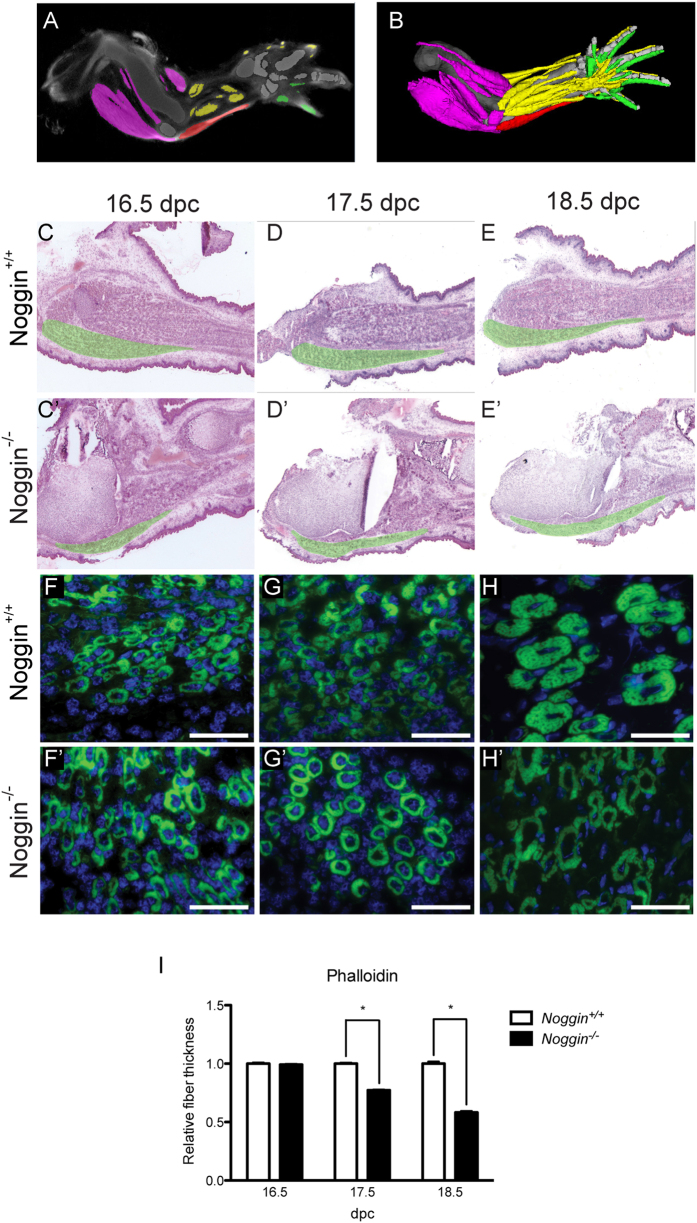
Analysis of the muscle fiber thickness. (**A,B**) The limb at 15–16 dpc using Jatlasviewer. The musculus flexor carpi ulnaris is colored in red. (**C–E’**) H&E staining on sagittal sections of the limbs at the indicated stages and genotype. The musculus flexor carpi ulnaris is digitally indicated in green. (**F–H’**) Actin immunofluorescence on cross-sections of muscles at the indicated stages. (**I**) Quantification using ImageJ of the thickness of the fiber. Values plotted as mean ± sem; over 100 fibers of at least 3 different mice embryos were analyzed per condition; *p < 0.05

The limb muscles of *Noggin*^−/−^ mice are strongly affected, with some of them missing and others severely malformed. Thus, to carry out a meaningful phenotypic analysis that would focus on the primary molecular differences rather than consequences of severe muscle malformation, we needed to select anatomically the same muscle in both genotypes. We performed H&E staining and compared the forelimb anatomy using the Jatlasviewer (www.emouseatlas.org/emap/analysis_tools_resources/software/jatlasviewer.html). We identified *musculus flexor carpi ulnaris* (indicated in red in Fig. 1A,B) as the muscle clearly identifiable and the least malformed in both wild type and *Noggin* null genotypes (Fig. 1C,E). For the analysis, we focused on three different developmental stages: one without an apparent defect (16.5 dpc), a dramatic defect (18.5 dpc) and the stage in between (17.5 dpc) (Fig. 1C–E’).

Since the histological appearance of the muscle suggested defective fiber thickness, we performed an F-Actin staining, using Phalloidin, and measured thickness of muscle fibers by measuring the muscle fiber diameter using ImageJ software. No difference in the overall morphology or in fiber thickness could be detected at 16.5 dpc in both genotypes. At 17.5 dpc *Noggin*^−/−^ myofibers were slightly but significantly thinner and this trend persisted until 18.5 dpc where the difference was apparent (Fig. 1F–H’). Quantitative analysis of this observation confirmed the histological phenotype (Fig. 1I).

### The loss of *Noggin* increases BMP signaling in embryonic muscles

**Figure 2:**
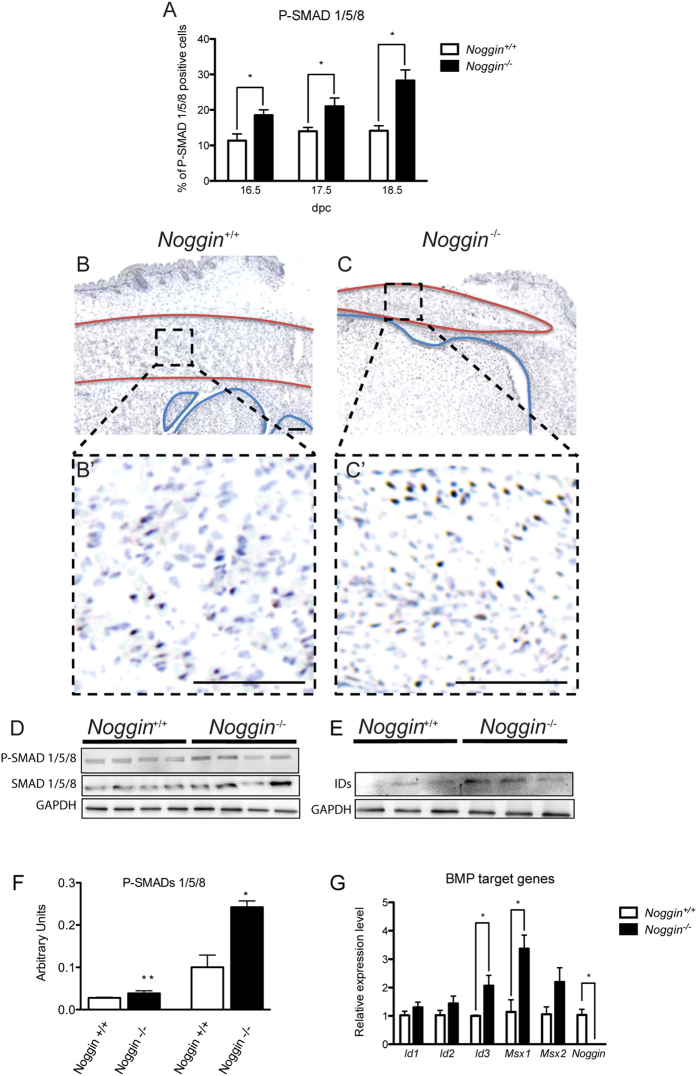
Analysis of the BMP signaling pathway. (**A**) Quantification of P-SMAD1/5/8 immunohistochemistry on sagittal sections of musculus flexor carpi ulnaris of *Noggin*^+/+^ and *Noggin*^−/−^ mice at the three different stages investigated. Values plotted as mean ± SEM; n = 5; *p < 0.05. Representative immunohistochemistry for P-SMAD1/5/8 at 16.5 dpc in *Noggin*^+/+^ (**B**) and *Noggin*^−/−^ muscle (**C**). Enlargement of muscle area delineated in black in B’ and C’. Muscle is delineated in red, cartilage and bone in blue. Scale bar: 100 μm. (**D**) WB analysis of the levels of P-SMAD1/5/8 compared to total SMAD1/5/8 and (**E**) IDs, normalized for GAPDH. (**F**) Quantification of the WB, data are representative of 3 independent experiments and values are expressed as mean ± sd; *p < 0.05, **p < 0.01. (**G**) qPCR analysis of BMP target genes at E16.5 dpc. Values plotted as mean ± SEM; n = 5; *p < 0.05.

Canonical BMP signaling phosphorylates SMAD1/5/8 and this phosphorylation status is used to detect active BMP signaling. Since Noggin is a BMP antagonist, its absence should result in augmented BMP signaling. Therefore we investigated the activation of the SMAD1/5/8 proteins. The immunohistochemical analysis for the phosphorylated SMAD1/5/8 (P-SMAD1/5/8) displayed an increase in the number of positive cells in the muscle already from 16.5 dpc onwards as quantified in Fig. 2A and shown in Fig. 2B–C’. To confirm that observation we carried out Western Blot (WB) analysis for both P-SMAD1/5/8 and IDs proteins levels (Fig. 2D,E respectively) in the same muscle. The results of the P-SMAD1/5/8 WB has been quantified (Fig. 2F).

To verify the presence of active BMP signaling in the *Noggin*^−/−^ *musculus flexor carpi ulnaris*, we investigated the expression of BMP immediate target genes. Using qRT-PCR at 16.5 dpc, we detected apparent upregulation of *Msx2* and significant upregulation of *Id3* and *Msx1* (Fig. 2G).

Thus, at 16.5 dpc we detected the onset of molecular changes preceding the histological defects detectable 24 hours later.

### The loss of *Noggin* does not affect the rate of proliferation

**Figure 3:**
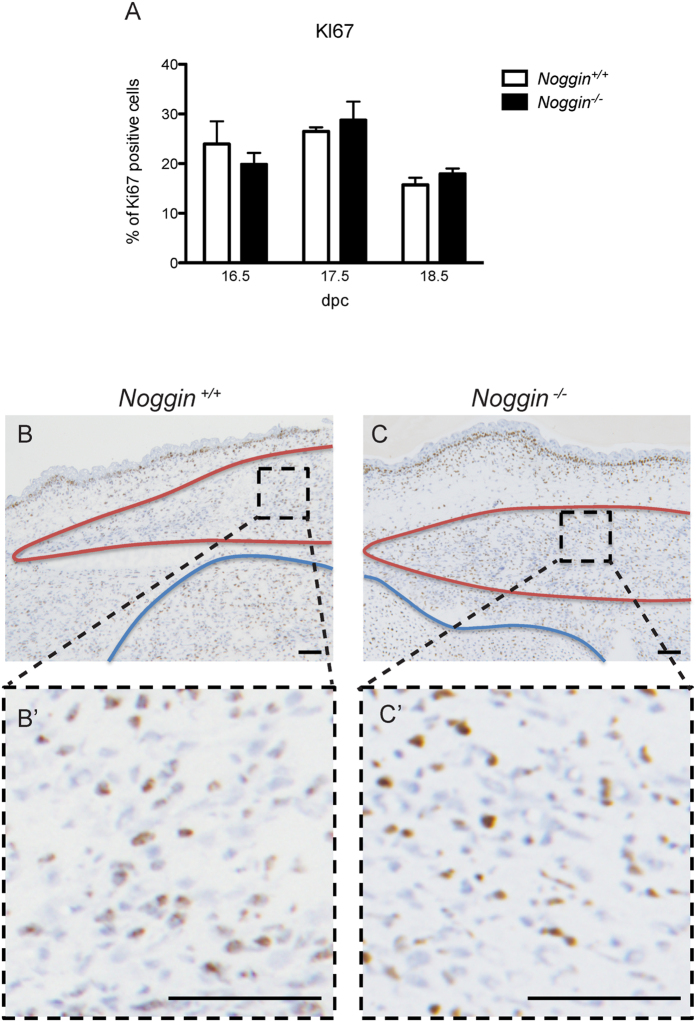
Analysis of the mitotic activity. (**A**) Quantification of KI67 immunohistochemistry on sagittal sections of musculus flexor carpi ulnaris of *Noggin*^+/+^ and *Noggin*^−/−^ mice at the three different stages investigated. Values plotted as mean ± SEM; n = 5; *p < 0.05. Representative immunohistochemistry for Ki67 at 16.5 dpc in *Noggin*^+/+^ (**B**) and *Noggin*^−/−^ (**C**) muscle. Muscle is delineated in red, cartilage and bone in blue. (B’–C’) Enlargement of muscle area delineated in black in **B** and **C**. Scale bar: 100 μm.

One of the functions of BMP signaling is to promote the exit from the cell cycle^15^. Thus one might expect that in the absence of Noggin, the cellular proliferation could be impaired leading to a decrease in the size of the muscle^16^. To address this issue, we analyzed the mitotic activity of the limb muscle cells using a KI67 antibody. KI67 is a nuclear protein present mainly in the G2, M, and at the end of the S phase and its presence is associated with active cell proliferation^17^. Despite the differences in muscle size and fiber thickness, no significant difference in cell proliferation could be detected in *Noggin*^−/−^ muscles between the 16.5 dpc (no observable muscle phenotype) and 18.5 dpc (a strong phenotype) (Fig. 3).

### *Noggin* ^−/−^ muscles have less Pax7^+^ progenitor cells

**Figure 4:**
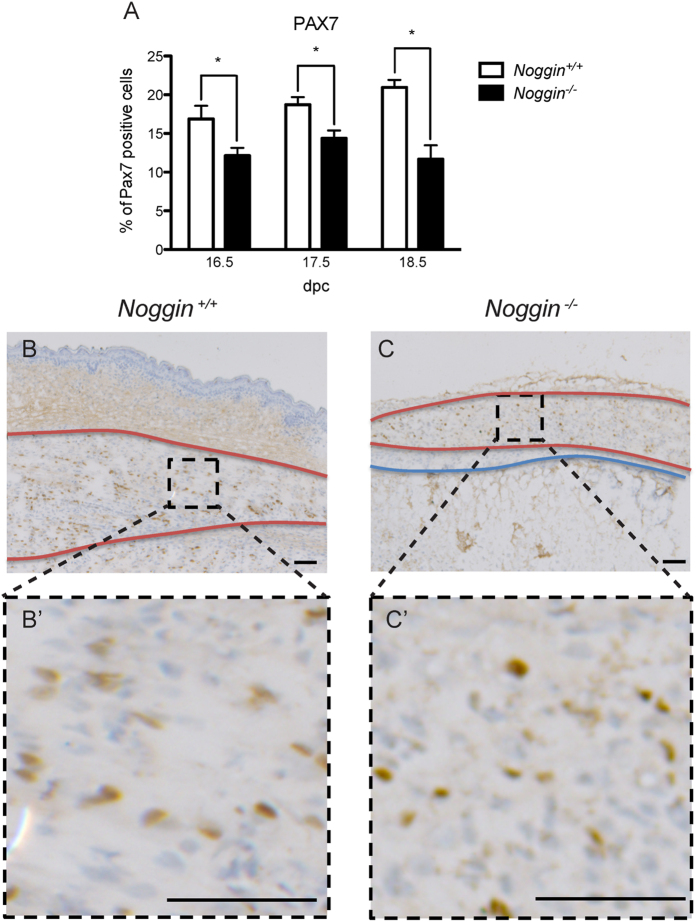
Analysis of the satellite cells. (**A**) Quantification of Pax7 immunohistochemistry on sagittal sections of musculus flexor carpi ulnaris of *Noggin*^+/+^ and *Noggin*^−/−^ mice at the three different stages investigated. Values plotted as mean ± SEM; n≤5; *p < 0.05. Representative immunohistochemistry for Pax7 at 18 dpc in *Noggin*^+/+^ (**B**) and *Noggin*^−/−^ (**C**) limbs. Muscle is delineated in red, cartilage and bone in blue. (**B’-C’**) Enlargement of muscle area delineated in black. Scale bar: 100 μm.

The cell proliferation was not affected in the muscle of *Noggin* null mice and the onset of muscle phenotype was relatively late during embryogenesis. Thus we hypothesized that perhaps not the muscle induction but rather muscle growth could be affected by excessive BMP signaling. During the muscle growth, Pax7^+^ cells provide the reservoir of stem cells that selectively enter myogenic differentiation and contribute to the growth of the muscle fiber during late embryonic development and in postnatal muscle repair processes^18^,^19^. Since BMP has a known negative effect on myogenesis (reviewed in ref. 20), we hypothesized that perhaps the number of Pax7^+^ cells in the late embryonic muscle could be reduced in the absence of Noggin. To investigate that, we carried out immunohistochemical analysis of the developing limb muscle for the expression of Pax7. Following the staining, we counted the number of positive cells using ImageJ software. Indeed, the number of Pax7^+^ cells was significantly reduced in *Noggin*^−/−^ muscles throughout the stages investigated (Fig. 4).

### Impairment of myogenic potential in *Noggin* null progenitors

**Figure 5:**
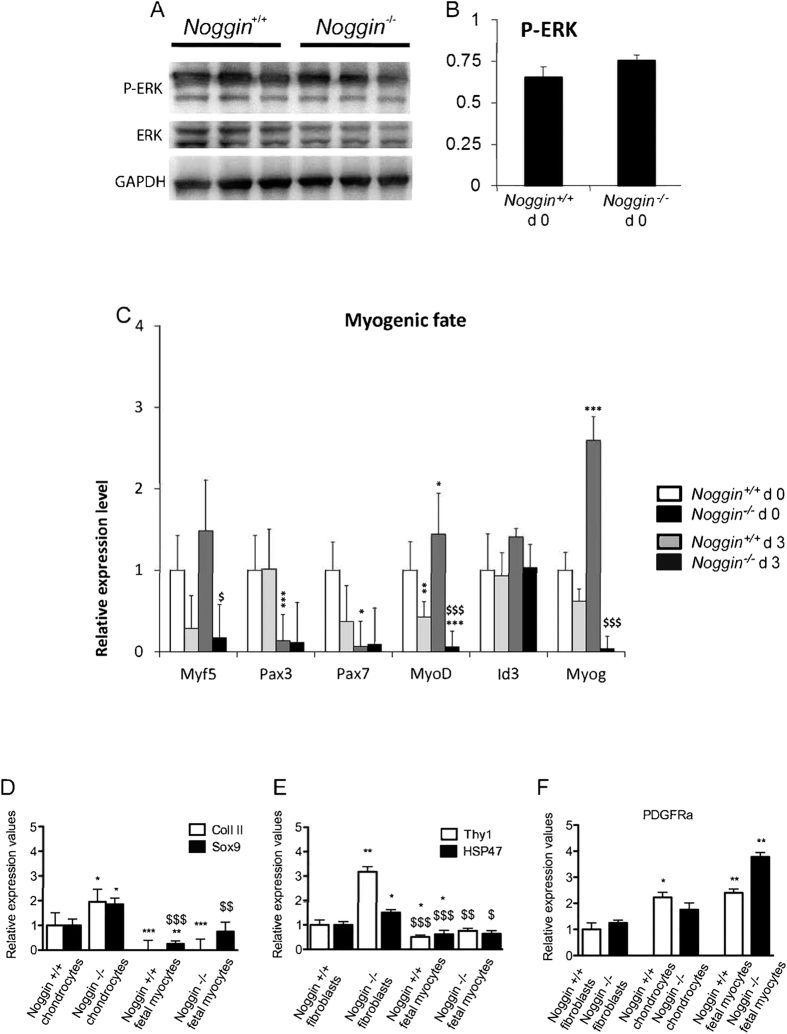
Myogenic differentiation of *Noggin*^−/−^ early fetal progenitors. **(A**) WB analysis and (**B**) protein quantification for the expression levels of P-ERK normalized on the total protein content and GAPDH for *Noggin*^−/−^ compared to *Noggin*^+/+^ 16.5 dpc muscle early fetal progenitors. (**C**) Expression levels of genes (*Myf5, Pax3, Pax7, Id3, MyoD* and *Myogenin*) involved in myogenesis at 0 and 3 days (d0, d3) of differentiation for *Noggin*^+/+^ and *Noggin−/−* cells. (**D**) Expression levels of chondrocytes (*Coll II, Sox9*), fibroblasts (*Thy1, Hsp47*) or mesenchymal stem cell (*Pdgfra*) in *Noggin*^+/+^ and *Noggin*^−/−^ early fetal progenitor cells. Results are expressed as ΔΔct normalized to specific housekeeping genes (*Gapdh, Hprt* and *Tbp*). Data are representative of 3 independent experiments and values are expressed as mean ± SEM; ^*^p < 0.05, ^**^p < 0.01, ^***^p < 0.001 Noggin^+/+^, ^$^p < 0.05, ^$$^p < 0.01, ^$$$^p < 0.001 Noggin^−/−^.

The cell proliferation was not affected in *Noggin*^−/−^ mice, but the number of Pax7^+^ progenitor cells was reduced. To exclude a defective proliferation, we analyzed the levels of P-ERK^21^,^22^. To accomplish that, fetal myoblast progenitors from *Noggin*^−/−^ and wild type embryos were isolated from 16.5 dpc limbs and cultured under myogenic conditions. No significant differences were detected in the levels of P-ERK, suggesting no defects in the proliferation rate were present in the muscle progenitors of *Noggin*^−/−^ mice (Fig. 5A,B).

Next, to address the differentiation potential of progenitor cells, *Noggin*^−/−^ cells were analyzed by qRT-PCR at 0 and 3 days of differentiation *in vitro* (Fig. 5C). At day 0, no significant differences in the expression levels of myogenic markers between *Noggin*^+/+^ and *Noggin*^−/−^ cells were detected, with the exception of *MyoD* mRNA. After 3 days in culture, the expression of the myogenic progenitor markers, *Pax3* and *Pax7*, decreased significantly irrespective of the genetic background. The expression levels of myogenic markers like *MyoD*, *Myf5* and *Myogenin* increased in the wild type while decreased significantly in the *Noggin*^−/−^ mouse cells (Fig. 5C). Thus as expected^23^,^24^, under the *in vitro* differentiation conditions used, the Pax3^+^ and Pax7^+^ cells did not maintain growth. At the same time, in the absence of *Noggin*, myogenic differentiation was repressed.

To ensure that the analyzed cell population was free of other cells that might affect the outcome of the experiment, we tested for the presence of cell types commonly contaminating such cultures, such as fibroblasts or chondrocytes. No large differences in the expression levels of *Collagen II (Coll II)* and *SOX9*^25^ (Fig. 5D), *Thy1*^26^ and *HSP47*^27^ (Fig. 5E), *PDGFRa*^28^ (Fig. 5F) were detected in the mRNA extracted from *Noggin*^+/+^ and *Noggin*^−/−^ cells, thus excluding any contamination of chondrocytes, fibroblasts or mesenchymal/endothelial stem cells respectively.

### Loss of myogenic differentiation in *Noggin* ^−/−^ progenitor cells

**Figure 6:**
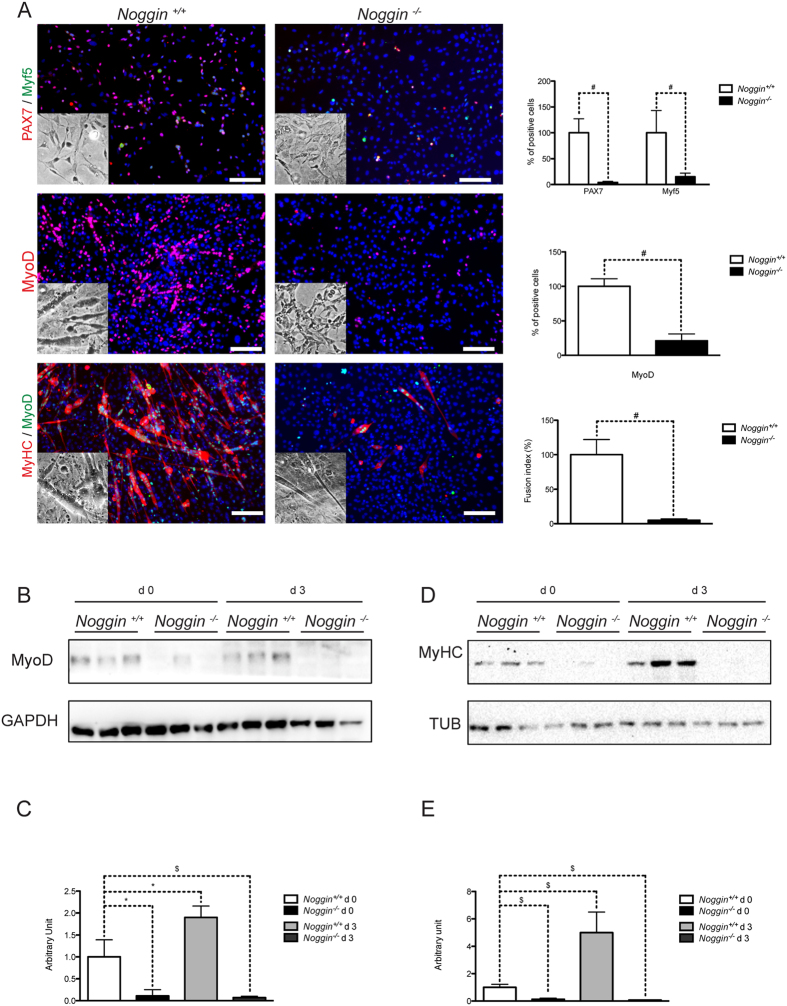
Quantification of myogenic differentiation of *Noggin*^−/−^ early fetal progenitors. (**A**) Upper panels: IF analysis for Pax7^+^ (red), Myf5^+^ (green) nuclei of early fetal myoblasts in growing medium (day 0 of differentiation) from both *Noggin*^+/+^ and *Noggin*^−/−^ embryos. Nuclei were stained with HOECHST (blue). The quantification of Pax7^+^ Myf5^+^ cells is reported in the upper right panel. Middle panels: IF analysis for MyoD^+^ (red) in *Noggin*^+/+^ and *Noggin*^−/−^ cells at day 0 of differentiation; histogram with the percentage of MyoD^+^ nuclei in *Noggin*^−/−^ cells with respect to *Noggin*^+/+^ cells are reported in the middle right panel. Lower panels: IF analysis for MyHC^+^ (red) myotubes and MyoD^+^ (green) nuclei in cells maintained during three days in differentiation medium. Fusion index (FI) analysis of differentiated *Noggin*^−/−^ cells compared to *Noggin*^+/+^ cells is shown in the right panel. Data are representative of 3 independent experiments and values are expressed as mean ± sd, relative to *Noggin*^+/+^; ^#^p < 0.001 vs *Noggin*^+/+^. An inset in each panel shows DIC images of cells. (**B**) WB analysis at 0 and 3 days of differentiation for MyoD; (**C**) protein quantification is reported in the lower panel. (**D**) WB analysis at 0 and 3 days of differentiation for MyHC; (**E**) protein quantification is reported in the lower panel. GAPDH and tubulin (TUB) were used as loading controls in B and C respectively. Data are representative of 3 independent experiments and values are expressed as mean ± sd; *p < 0.05, ^$^p < 0.01.

Next, we analyzed the cellular distribution of Pax7 and Myf5. Dual immunofluorescence revealed an apparent decrease in number of nuclei positive for Pax7 and Myf5 in *Noggin*^−/−^ fetal myoblasts as compared to *Noggin*^+/+^. The percentage of Pax7^+^ and Myf5^+^ nuclei in *Noggin*^−/−^ cultures was reduced to respectively 4% ± 2.26 and 15.8% ± 7.8 of *Noggin*^+/+^ values (Fig. 6A). After three days under the differentiation condition the number of MyoD^+^ cells was significantly reduced in *Noggin*^−/−^ fetal myoblasts as compared to controls (8% ± 4 of *Noggin*^+/+^ cells; Fig. 6A). The myoblasts during the later stages of differentiation fuse to form myotubes (Suppl Fig. 1A). Thus we determined the fusion index calculated as the percentage of nuclei inside of MyHC^+^ and α-Sarcomeric Actinin^+^ myotubes to the number of total nuclei of *Noggin*^−/−^ cultures and showed that it was also markedly decreased (Fig. 6A and Suppl. Fig. 1B).

To semi-quantify these observations we carried out a protein expression analysis by WB. While MyoD and MyHC protein levels were detectable in *Noggin*^+/+^ cells, they were almost undetectable in *Noggin*^−/−^ cells at early (d0) and late (d3) stage of differentiation (Fig. 6B–E).

In addition, the levels of embryonic MyHC (eMyHC) strongly expressed in *Noggin*^+/+^ cells late stage, were barely detectable in *Noggin*^−/−^ cells at both time points of differentiation (Suppl Fig. 1C).

### Aberrant adipogenic potential in *Noggin* ^−/−^ progenitors

**Figure 7:**
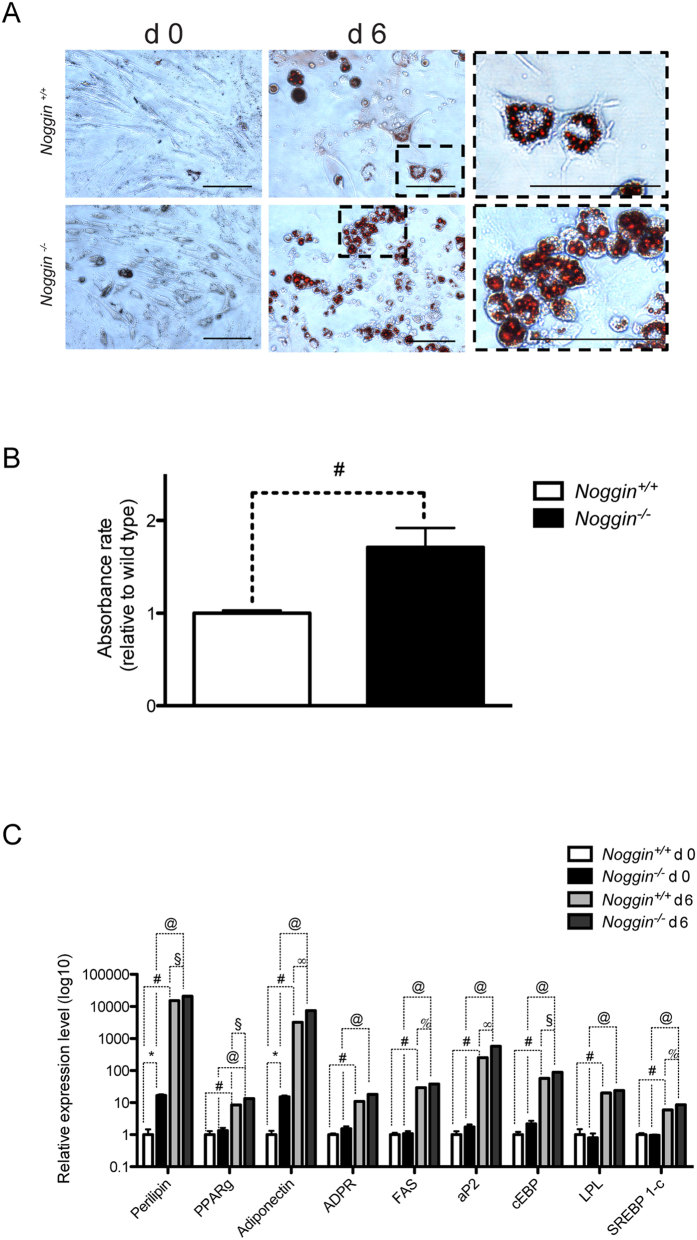
Adipogenic induction of *Noggin*^−/−^ early fetal progenitors. (**A**) Oil-Red-O staining of *Noggin*^+/+^ (upper panels) and *Noggin*^−/−^ (lower panels) early fetal progenitors cultivated for 6 days under adipogenic condition; higher magnifications of the inset are reported in right panels. (**B**) Lipid quantification of *Noggin*^+/+^ and *Noggin*^−/−^ cells. (**C**) qRT-PCR analysis of adipogenic markers at d0 and d6 of differentiation. Data of at least 5 independent experiments are expressed as ΔΔct values normalized to specific housekeeping genes (*Gapdh, Hprt and Tbp*); values reported as mean ± SEM, normalized to *Noggin*^+/+^ values. ^*^p < 0.01, ^#^p < 0.001; @ p < 0.001; ^∞^p < 0.05, ^§^p < 0.01, % p < 0.001.

It has been reported that BMP signaling pathway is also involved in adipocyte commitment and differentiation *in vitro* and *in vivo*^29^,^30^. To investigate this aspect of cellular differentiation in *Noggin*^−/−^ cells, we tested their adipocyte commitment *in vitro* by growing them in adipogenic medium. *Noggin*^+/+^ progenitor cells were able to shift towards an adipocyte fate, even if they were still able to generate myotubes (Suppl Fig. 1D). *Noggin*^−/−^ cells, however, only differentiated towards adipocytes, even more efficiently compared to *Noggin*^+/+^ cells (1.7 ar ± 0.2 vs. 1 ar ± 0.03) as shown by quantification of Oil-Red-O staining (Fig. 7A,B).

The molecular signature of these cells further supported their adipogenic phenotype. *Noggin*^−/−^ cells showed a significant increase in the expression of the majority of the adipocyte genes after 6 days of adipogenic induction as shown by qRT-PCR data (Fig. 7C and Table 1- please note the logarithmic scale on the Figure). Notably, at day 0 Perilipin (166% ± 1.5) and Adiponectin (151% ± 1.6) were already present at increased levels as compared to the *Noggin*^−/−^ cells (Fig. 7C).

## Discussion

We have previously reported on a muscle defect in *Noggin*^−/−^ mice^4^. Here we investigated the molecular basis of that phenotype. Interestingly, the defect has a late onset, indicating that the initial muscle induction and formation occurs normally. At the later stages however, when the muscle acquires more mass, it becomes sensitive to the absence of *Noggin*. Our analysis indicates that the loss of *Noggin* in the mouse embryonic muscle resulted in an increased BMP signaling together with a decrease in the relative number of Pax7^+^ cells. Intriguingly, the differentiation defect persisted in *in vitro* setting suggesting that it was cell autonomous. Specifically the cells lost the ability to enter the myogenic pathway while maintained the adipogenic one.

Our analysis shows that the loss of *Noggin* during mouse embryonic development results in an increased BMP signaling in developing muscle. Molecularly it has been shown that the inhibitory effect of BMP signaling on the myogenic program is a result of the sequestering of the E-proteins by the immediate BMP target genes (*Id1, 2, 3*)^31^,^32^. Thereby the ID proteins block the MRF-mediated activation of the myogenic program, which is dependent on the binding of the MRFs to these E-proteins. Furthermore, Msx1 was shown to block cellular differentiation by preventing cell cycle exit and to antagonize the myogenic activity of Pax3 in migrating limb muscle precursors^33^,^34^. Since the BMP target genes are upregulated in the *Noggin*^−/−^ mice, the sequestering of the E-proteins, the inhibition of cell cycle exit and/or the loss of differentiation of the migrating limb precursors are all potential mechanisms underlying the *Noggin*^−/−^ muscle phenotype.

The BMP signaling was shown to regulate the patterning of the somite and the myogenic program^3^, thus a muscle defect in BMP antagonist mutants could have been anticipated. However, the extent of the muscle defect in the *Noggin*^−/−^ mice and the late onset was far greater than reported for other BMP antagonist mutants^35^. Reshef *et al*. did suggest that the epaxial musculature in the *Noggin*^−/−^ mice was largely absent in the posterior part of the embryo, but no further research was conducted on the muscles of the *Noggin*^−/−^ mice^36^. The work presented by Lassar’s group did in fact lay a foundation for our work. Reshef *et al*. have shown that the precise regulation of BMP signaling by Noggin and other antagonists, controls the entry of Pax3^+^ cells into myogenic differentiation program defined by the expression levels of MyoD and Myf5. This regulatory loop is therefore essential for the myogenesis within the somites *in vitro* and *in ovo*. We have extended the work of Reshef *et al*. by showing that the same regulatory loop used also at later stages of development in mouse. Specifically, we show that, in *Noggin*^−/−^ embryos, not only the muscle fibers exhibit a morphological defect but the maintenance of the fetal myogenic progenitor pool is impaired as well. In fact, we show for the first time that *Noggin*^−/−^ myogenic progenitors are unable to properly differentiate into the myogenic lineage, but display a more efficient commitment to adipogenic lineage.

*In vitro*, BMP signaling was shown to balance the proliferation and differentiation of muscle satellite cells. Upon differentiation, BMP signaling is downregulated resulting in the activation of the myogenic program and formation of myofibers^37^,^38^. In contrast, when BMP was applied ectopically in the chicken wing, the number of muscle fibers and Pax7^+^ cells increased^14^,^16^. However, when BMP was applied during earlier stages of chicken development, similar chondrogenesis and myogenesis defects were seen as in the *Noggin*^−/−^ mice^39^. These results strongly suggest that the timing and the localization of the BMP signal and the differentiation status of the target cells determines the molecular response explaining the apparent contradiction in the results published by us and among these research groups.

It was suggested that cells in the somitic environment could have different intrinsic properties as they respond differently to the same signals^40^. For example, embryonic myoblasts were shown to be insensitive to BMP signaling allowing them to stop proliferating and differentiate into the primary myofibers. For fetal myoblasts and satellite cells, however, it was shown that the level of BMP signaling has to be reduced, by either reducing the expression of BMP ligands, or antagonizing the signaling, in order to let differentiation and fusion into secondary fibers occur^16^. This potentially explains the *Noggin*^−/−^ muscle phenotype, as the increased BMP signaling in the muscle would have no effect on the differentiation of the primary fibers, but it could inhibit the generation of the secondary fibers. Therefore fetal myoblasts, which normally give rise to the secondary fibers, maintain the committed but undifferentiated state. Indeed, we observe mononucleated myoblasts in the *Noggin*^−/−^ muscle, concomitant with a reduction of multinucleated myofibers. The limited number of primary fibers that is formed in the absence of *Noggin* results in a muscle that fails to maintain its organization.

Another explanation of the observed phenotype could be based on a possibility of lineage switch. In fact, the myogenic potential of C2C12 cells can be fully blocked by the addition of BMP ligands, which then promote osteochondrogenic differentiation of this cell line^41^,^42^ and reviewed in ref. 43. Thus we hypothesize that a number of muscle progenitor cells are drawn into the chondrogenic lineage in the *Noggin*^−/−^ limb^44^,^45^. This would deplete the pool of muscle precursors in a way that initially enough cells are left to maintain the muscle organization, but at later stages, when the muscles grow rapidly, there are not enough cells to supply the muscle. In this view, the loss of *Noggin* induces SMAD-dependent increase in BMP signaling that results in an inhibition of fetal myoblast differentiation and a reduction in the number of satellite cells, which causes a severe late-onset muscle defect. At the same time, the prechondrogenic pool increases, leading to excessive chondrogenesis.

To test this hypothesis we investigated the molecular and cellular properties of mononuclear myogenic progenitors isolated from *Noggin*^+/+^ and *Noggin*^−/−^ littermates. Interestingly, the transcript levels of early myogenic markers (Pax3 and Pax7) had the same dynamics in *Noggin*^+/+^ and *Noggin*^−/−^ progenitors. However, protein levels of Myf5 and MyoD were significantly reduced in *Noggin*^−/−^ progenitors after 3 days of myogenic induction. Indeed, it has been previously reported that no significant differences were detected for Pax3, Pax7 and Myf5 expression in *Noggin*^−/−^ embryonic muscles^13^. The regulation of Pax7 protein levels is complex and not yet completely defined. In principle we cannot exclude that in our conditions, as recently reported^46^ and reviewed in ref. 23, a post-translational modification could induce the ubiquitination and consequently a cleavage of Pax7.

In this view a complete inhibition of the MyoD and possibly Myogenin proteins is probably the main cause of the lack in differentiation of early fetal myoblasts in our cultures as it was previously reported^47^.

One of the key findings of this work is that MyoD and consequently embryonic and adult MyHC protein levels are strongly decreased in *Noggin*^−/−^ progenitors. Recently, the pattern of expression of MyHC isoforms has been fully characterized and defined as a transition form from one cohort of MyHC isoforms (embryonal, neonatal and type I MyHC) to another (fast type II isoforms), occurring during muscle cell differentiation^48^. Thus, eMyHC dramatically decreased in *Noggin*^−/−^ cells at both time points considered, could not support further expression of the adult MyHC isoform.

Intriguingly, the response of the *Noggin*^−/−^ muscle progenitors to adipocytogenic conditions dramatically differs from the wild type cells. The absence of Noggin and thus overexposure to BMP signaling during embryonic development induces the muscle progenitor cells to be significantly more responsive to adipogenic activation than the corresponding wild type cells. Indeed, *Adiponectin* and *Perilipin* genes normally expressed in adult white adipose tissue^49^, are also highly transcribed in these aberrant progenitor cells.

These results are in line with previous reports showing the participation of BMP4 in adipocyte lineage commitment and the rescue induced with Noggin exposure *in vivo*^50^. Moreover, it has been recently demonstrated that murine myoblasts (C2C12) display lipid accumulation upon BMP6 stimulation^51^.

Taken together our results stress the importance of balance between Noggin and BMPs for myogenic commitment of early progenitor cells during limb development. The absence of *Noggin* could induce a concomitant BMP over-signaling, likely responsible for the impaired myogenic commitment of *Noggin*^−/−^ progenitors. Indeed, BMP2, in cooperation with SMAD1/4 and C/EBPα, can induce the expression of PPARγ2, key transcription factor for adipocyte differentiation^52^. Our data also indicate the power of cell memory, where the level of BMP exposure is remembered during later differentiation stages, even *in vitro*, and even when the actual signal is not present anymore.

Further studies are necessary to define the role of other BMP inhibitors^53^, e.g. Chordin or Follistatin, Xnr3 or Cerberus, which could eventually compensate the absence of Noggin and be involved in the aberrant differentiation ability observed in *Noggin*^−/−^ muscle progenitors^54^. It could be also interesting to investigate if the alteration of Noggin/BMP balance is involved in the adipogenic conversion of skeletal muscle precursors observed in chronic muscle degenerations.

## Materials and Methods

### Embryo processing

All the mouse experiments were carried out in accordance with European guidelines on animal research and approved by the ethics committee at the University of Leuven. *Noggin* heterozygous mice were maintained in CD1 background as described before^4^. Mice were sacrificed and embryos were collected at 16.5, 17.5 and 18.5 days post coitus (dpc). Forelimbs were isolated using forceps and subsequently embedded in Tissue-Tek (Laborimpex), snap frozen in liquid nitrogen and stored at −80 °C. Hind limbs were processed for beta-galactosidase (beta-gal) genotyping. Frozen sections (5 μm) for immunological and histological staining (Haematoxylin and eosin; standard protocol) were made using a cryostat (Prosan).

### Beta-galactosidase genotyping

Targeted *Noggin* inactivation was achieved by replacing *Noggin* exon with LacZ expression cassette^3^ permitting the genotyping by beta-gal staining. The well-described phenotypic differences were used to discriminate between heterozygous and homozygous animals (4,55). Hind limbs were fixed in 4% PFA in PBS for 10 minutes and washed twice with LacZ1 solution (PBS, 2mM MgCl_2_, 5 mM EGTA) for 10 minutes at RT, followed by another washing step with LacZ2 (PBS, 0.1 M Na_3_PO_4_, 2 mM MgCl_2_, 0.01% sodium deoxycholate, 0.02% NP-40; pH 7.2–7.4) for 5 minutes at RT. Staining was performed by applying staining solution (5 mM K_3_Fe(CN)_6_, 5 mM K_4_Fe(CN)_6_, 0.5 mg/ml, X-gal in LacZ2 buffer for 2–3 hours at 37 °C or ON at RT. After washing steps with PBS and clearing with 2% KOH, limbs were treated with an increasing gradient of glycerol/KOH before storage.

### Immunohistochemistry

Frozen sections were fixed with methanol for 10 minutes at −20 °C. After quenching in 3% H202/MQ and washing in TBST, antigen retrieval was performed with sodium citrate buffer (pH6), followed by additional washing with TBST. Sections were blocked with Normal Goat Serum (Millipore) (1/5 in TBST) and O/N incubated with the primary antibody (P-SMAD1/5/8, Cell Signaling, 9511S; 1/100) at 4 °C. After washing, the biotinylated secondary antibody was applied for 30 minutes at RT. The signal was amplified with the ABC reagent (Vectastain, dilution according to manufacturer’s protocol) for 30 minutes at RT. Phalloidin staining was performed using manufacturers protocol (Sigma). After washing, the color was developed using DAB (DAKO, dilution according to manufacturer’s protocol) and sections were mounted using Pertex.

The P-SMAD1/5/8 protocol was used with following modifications. Frozen sections were fixed with ethanol and methanol for 5 minutes and quenched in 0.5% H202/MeOH. Washing steps were performed with PBS, a KI67 antibody (DAKO TEC3; 1/100) or Pax7 antibody (Hybridoma bank, Iowa, USA; 1/400) was used and blocking was done with 10%NGS/PBS. ImageJ software was used to measure muscle fiber thickness and the quantification of the percentage of P-SMAD1/5/8, KI67 or Pax7^+^ cells. The significance of the difference in expression was analyzed using 2way ANOVA test with Bonferroni comparison and significance was achieved with p-values less than 0.05 (Student’s T-test).

### Gene expression analysis

For muscle tissue, RNA was isolated by scraping the muscles of at least 10 frozen sections 16.5 dpc embryonic limbs using the High Pure RNA Isolation Kit (Roche Applied Science). At least three RNA isolations were performed per condition and subsequently reverse transcribed using Primescript RT Reagent (Takara; manufacturers protocol). qRT-PCR was performed in duplicate with gene specific primers (Table 2) with SYBR Premix Ex Taq II (Takara; manufacturers protocol) using Rotor-gene 6000 detection system (Corbett Research, Westburg). Gene expression was normalized to the housekeeping gene *Hprt1* and presented as a ratio to control embryos. The significance of the difference in expression was analyzed using the Student’s T-test.

For the cells, *Noggin*^+/+^ and *Noggin*^−/−^ early fetal myoblasts, chondrocytes and fibroblasts were used for RNA extraction with RNA Mini Kit, removing genomic DNA traces by Turbo DNase. 1ug RNA was reverse-transcribed with SuperScript III kit and qPCR was performed in 384 well plates (10ul final volume; thermal profile, 95 °C 15″−60 °C 45″ (40x); ViiA 7 qPCR plate reader), using Platinum Sybr Green Mix, 1ul 1:4 diluted cDNA and 250 nM primers (all kits, reagents and plate reader by Life Technologies, Carlsbad, CA, USA). Proliferating MEF cells as well as fibroblasts from adult mice or 3T3-L1 preadipocytes (ATCC, Manassas, VA, USA) were used as positive controls.

### Fetal myoblasts isolation and myogenic differentiation

Early fetal myoblasts were isolated from forelimbs of E16.5 *Noggin*^−/−^ and control embryos (after careful removal of bones and skin) as previously described^40^. Briefly, tissues were dissociated one hour at 37 °C with 0.06% Collagenase (from Clostridium Histolyticum, Sigma-Aldrich St. Louis, Mo, USA, for all reagents excepted when notified) and 0.04% Pancreatin in PBS (Gibco-Invitrogen, Carlsbad, CA, USA). After centrifugation, cells were seeded in collagen-coated dishes in presence of Dulbecco’s modified Eagle’s medium (DMEM)-high glucose and 20% fetal bovine serum (FBS; Gibco-Life Technologies, Carlsbad, CA, USA), supplemented with 1% Chicken Embryo Extract (Sera Laboratories International, UK), 100 mg/ml Sodium Pyruvate, 100 IU/ml penicillin and streptomycin, 2 mM L-glutamine. The plates were incubated at 37 °C, 5% CO_2_, 5% O_2_ and the medium refreshed every two days. When cells reached confluence, the differentiation was induced by shifting medium to DMEM high glucose supplemented with 2% horse serum (Gibco-Invitrogen, Carlsbad, CA, USA) and 100 mg/ml Sodium Pyruvate (differentiation medium; DM).

### Adipogenic differentiation

Early fetal myoblasts were dispended on collagen coated dishes and expanded till confluence, in presence of STEMPRO Adipogenesis Differentiation Medium (Gibco-Lyfe Technologies, Carlsbad, CA, USA) to enhance adipogenesis, as described from the manufacturer. 3T3-L1 preadipocytes (ATCC) used as control for adipocyte differetiation were grown in DMEM supplemented with 10% (v/v) fetal calf serum (Gibco-Lyfe Technologies, Carlsbad, CA, USA), 25 mM HEPES (pH 7.0), 100 U/ml penicillin and streptomycin, and 25 μg/ml Fungizone Antimycotic (Gibco-Lyfe Technologies, Carlsbad, CA, USA). To induce adipocyte differentiation, confluent cells were cultured in DMEM with 10% (v/v) FBS (Gibco-Lyfe Technologies, Carlsbad, CA, USA), 25 mM HEPES (pH 7.0), 100 U/ml penicillin and streptomycin, and 25 μg/ml fungizone, plus 0.5 mM isobutylmethyl-xanthine, 1 μM dexamethasone, and 1 μg/ml insulin (MDI). MDI was replaced 48 h after, with insulin (1 μg/ml), and the medium changed every two days.

### Immunofluorescence analysis

Cells at day 0 and 3 of differentiation were fixed with 4% PFA (Polysciences Europe GmbH, Germany) and permeabilized with 0.2% PBS-Triton, 1% Bovine Serum Albumin (BSA). A blocking solution containing donkey serum was applied and primary antibodies were incubated overnight (O/N) in PBS supplemented with 2.5% w/v BSA. Primary antibodies: 1:50 anti-rabbit Myf5 (c-20, Santa-Cruz Byotechnology, Heidelberg, Germany), 1:3 mouse anti-PAX7 (Developmental Studies Hybridoma Bank, DSHB, Iowa, USA), 1:50 mouse anti-MyoD (Dako, Agilent Technologies, Denmark), 1:30 rabbit anti-MyoD (Santa Cruz Byotechnology, Heidelberg, Germany), 1:10 mouse anti-MyHC (clone MF20, Developmental Studies Hybridoma Bank, DSHB, Iowa, USA), anti-mouse α-Sarcomeric Actinin (Abcam, Cambridge, UK). Secondary Alexa Fluor donkey antibodies (Life Technologies, Carlsbad, CA, USA) were diluted at 4 μg/ml in PBS supplemented with 2.5% w/v BSA and the nuclei counterstained with 10 ug/ml Hoechst. Fluorescence images were acquired at 10x magnification via Eclipse Ti inverted microscope (Nikon). Fusion index (number of myotube nuclei vs total number of nuclei) such as the percentage of myotube positive for a specific marker were calculated for a minimum of four random fields from *Noggin*^+/+^ and *Noggin*^−/−^ embryo cells of different embryos (n = 7, three different litters).

### Western blot (WB) analysis

WB analysis were performed on cell lysates in RIPA buffer supplemented with 10 mM Sodium Fluoride, 0.5 mM Sodium Orthovanadate, 1:100 Protease Inhibitor Cocktail and 1 mM Phenylmethanesulfonyl Fluoride. Equal amounts of protein (30 μg) were heat-denatured in sample-loading buffer (50 mM Tris-HCl, pH 6.8, 100 mM DTT, 2% SDS, 0.1% bromophenol blue, 10% glycerol), resolved by SDS-PAGE and transferred to nitrocellulose membranes (GE Healthcare Bio-Sciences, Pittsburg, USA). The filters were blocked with Tris-buffered saline (TBS) containing 0.05% Tween and 5% non-fat dry milk and then incubated overnight with the different primary antibodies: rabbit 1:1000 anti-P-SMAD1/5/8 (Cell Signaling); rabbit 1:100 anti-SMAD1/5/8 (ThermoScientific); rabbit 1:100 anti-IDs (SantaCruz); rabbit 1:100 anti-P-ERK (SantaCruz); mouse 1:200 anti-ERK (SantCruz); mouse 1:100 anti-MyoD (Dako, Agilent Technologies, Denmark); mouse 1:10 anti-MyHC and mouse 1:5 anti-eMyHC (Developmental Studies Hybridoma Bank, DSHB, Iowa, USA), mouse 1:1000 anti-Tubulin beta, clone KMX-1 (Millipore, Chemicon, Billerica, MA, USA), anti-rabbit GAPDH. All secondary horseradish peroxidase (HRP)-conjugated antibodies (Santa Cruz Biotechnology, CA, USA) were diluted 1:5000 in TBS-Tween and 2.5% non-fat dry milk. After incubation with SuperSignal Dura Chemiluminescence substrate (Thermo Scientific, Waltham, MA USA), WB analysis was performed with GelDoc chemiluminescence detection system (BioRad, Temse, Belgium). Quantification by relative densitometry was obtained normalizing versus background and GAPDH or Tubulin, using the QuantityOne software (BioRad).

### Oil-Red-O staining

Lipid accumulation in early fetal myoblasts was assessed by Oil-Red-O staining. Briefly, cells were fixed for 15 min in 4% PFA, washed three times in PBS, and stained for 30 min with the lipophilic dye Oil-Red-O in 65% isopropanol, followed by washing steps and drying. Bright field Oil-Red-O images were captured at 10x and 20x magnification using an Eclipse Ti inverted microscope (Nikon). Lipids extracted with a petrol ether/isopropanol mixture (3:2) were quantified for their absorbance at 490 nm with Victor spectrophotometer (PerkinElmer, Massachussets, USA).

### Statistical analysis

All results were expressed as mean ± SD, with the exception of gene expression (mean ± SEM). Representative WB show independent samples. When two groups were compared, a Student’s t-test was used. When three or more groups were compared a two-way ANOVA was used. The statistical significance of the differences between percentage values was assessed using a Kruskal-Wallis one-way analysis of variance by ranks test. All statistical tests were performed via Prism software (GraphPad).

## Additional Information

**How to cite this article**: Costamagna, D. *et al*. Noggin inactivation affects the number and differentiation potential of muscle progenitor cells *in vivo*. *Sci. Rep*. **6**, 31949; doi: 10.1038/srep31949 (2016).

## Supplementary Material

Supplementary Figure S1

## Figures and Tables

**Table 1:** ΔΔct values normalized to specific housekeeping genes of qRT-PCR analysis reported in Fig. 7C.

		Perilipin	PPARg	Adipo-nectin	ADPR	FAS	aP2	cEBP	LPL	SREBP1
*Noggin*^+/+^	d0	1.0 ± 0.5	1.0 ± 0.3	1.0 ± 0.3	1.0 ± 0.1	1.0 ± 0.1	1.0 ± 0.3	1.0 ± 0.2	1.0 ± 0.5	1.0 ± 0.1
*Noggin*^−/−^	d0	16.6 ± 1.5**	1.3 ± 0.3	15.2 ± 1.6**	1.5 ± 0.3	1.1 ± 0.2	1.7 ± 0.3	2.2 ± 0.5	0.8 ± 0.2	0.9 ± 0.1
*Noggin*^+/+^	d6	15.2 × 10^3^ ± 0.4***	8.5 ± 0.3***	3.2 × 10^3^ ± 0.4***	10.9 × 10^3^ ± 0.3***	29.4 ± 0.2***	253.8 ± 0.4***	57.4 ± 0.2***	20.1 ± 0.2***	5.9 ± 0.0***
*Noggin*^−/−^	d6	20.7 × 10^3^ ± 0.3*** $$ °°°	13.5 ± 0.2*** $ °°°	7.4 × 10^3^ ± 0.3*** $ °°	17.9 ± 0.4*** °°°	38.1 ± 0.4*** $$$°°°	569.2 ± 0.1*** $ °°°	88.5 ± 0.5*** $$ °°°	23.9 ± 0.3*** °°°	8.5 ± 0.4*** $$$ °°°

**Table 2:** list of primers.

Gene	Forward primer (5′–3′)	Reverse primer (5′–3′)
*Adiponectin*^56^	GGAGAGAAAGGAGATGCAGGT	CTTTCCTGCCAGGGGTTC
*ADRP*^57^	CTTGTGTCCTCCGCTTATGTCAGT	CTGCTCCTTTGGTCTTATCCACCA
*aP2*^58^	GGAAGCTTGTCTCCAGTGAA	GCGGTGATTTCATCGAATTC
*C/EBPα*^58^	TGCGCAAGAGCCGAGATAAA	CCTTGACCAAGGAGCTCTCA
*Coll II*	CCAGGATGCCCGAAAATTAG	TTCTCCCTTGTCACCACGAT
*FAS*^59^	AGTGTCCACCAACAAGCG	GATGCCGTCAGGTTTCAG
*GAPDH*^60^	TGGTGAAGGTCGGTGTGAAC	GCTCCTGGAAGATGGTGATGG
*HPRT*^60^	TGGATACAGGCCAGACTTTGTT	CAGATTCAACTTGCGCTCATC
*Hprt1*	TGCTGACCTGCTGGATTACA	TATGTCCCCCGTTGACTGAT
*Hsp 47*^61^	GCAGCAGCAAGCAACACTACAACT	AGAACATGGCGTTCACAAGCAGTG
*Id1*	GAGTCTGAAGTCGGGACCAC	AACACATGCCGCCTCGG
*Id2*	CCTGCATCACCAGAGACCTG	GGGAATTCAGATGCCTGCAA
*Id3*^62^	TGCTACGAGGCGGTGTGCTG	TGTCGTCCAAGAGGCTAAGAGGCT
*LPL*^63^	CTGCTGGCGTAGCAGGAAGT	GCTGGAAAGTGCCTCCATTG
*Msx1*	GCCAGAAGATGCTCTGGTGA	TCAGCCGTCTGGCTGGG
*Msx2*	GCCTCGGTCAAGTCGGAAAA	GGCTCATATGTCTGGGCGG
*Myf5*^64^	ACAGAGCTGCTCTGAGCCCACC	TACATCAGGACAGTAGATGCTGTC
*MyoD*^65^	GAGCAAAGTGAATGAGGCCTT	CACTGTAGTAGGCGGTGTCGT
*Myog*^65^	ATGGAGCTGTATGAGACATCCCC	CGACACAGACTTCCTCTTACAC
*Noggin*	GGGGCGAAGTAGCCATAAAG	GGGGCGAAGTAGCCATAAAG
*Pax3*^64^	ACTACCCAGACATTTACACCAGG	AATGAGATGGTTGAAAGCCATCAG
*Pax7*^65^	GACGACGAGGAAGGAGACAA	ACATCTGAGCCCTCATCCAG
*PDGFRa*^65^	CCTGTAACTGACACGCTCCG	CCATTTCCAAACCGCACA
*Perilipin*^58^	TTGGGGATGGCCAAAGAGAC	CTCACAAGGCTTGGTTTGGC
*PPARγ*^66^	GCCCTTTGGTGACTTTATGGA	GCAGCAGGTTGTCTTGGATG
*Sox9*^67^	CAAGAACAAGCCACACGTCA	TGTAATCGGGGTGGTCTTTC
*SREBP-1c*^59^	CTGGAGACATCGCAAACAAGC	ATGGTAGACAACAGCCGCATC
*TBP*^60^	CAAACCCAGAATTGTTCTCCTT	ATGTGGTCTTCCTGAATCCCT
*Thy 1*^68^	GCCGCCATGAGAATAACA	GCTAGGGTAAGGACCTTGAT
