# Supplementary figures and images for: Noggin inactivation affects the number and differentiation potential of muscle progenitor cells in vivo
Source: Sci Rep. 2016 Aug 30;6:31949. doi: 10.1038/srep31949 (PMC5004166; doi:10.1038/srep31949)

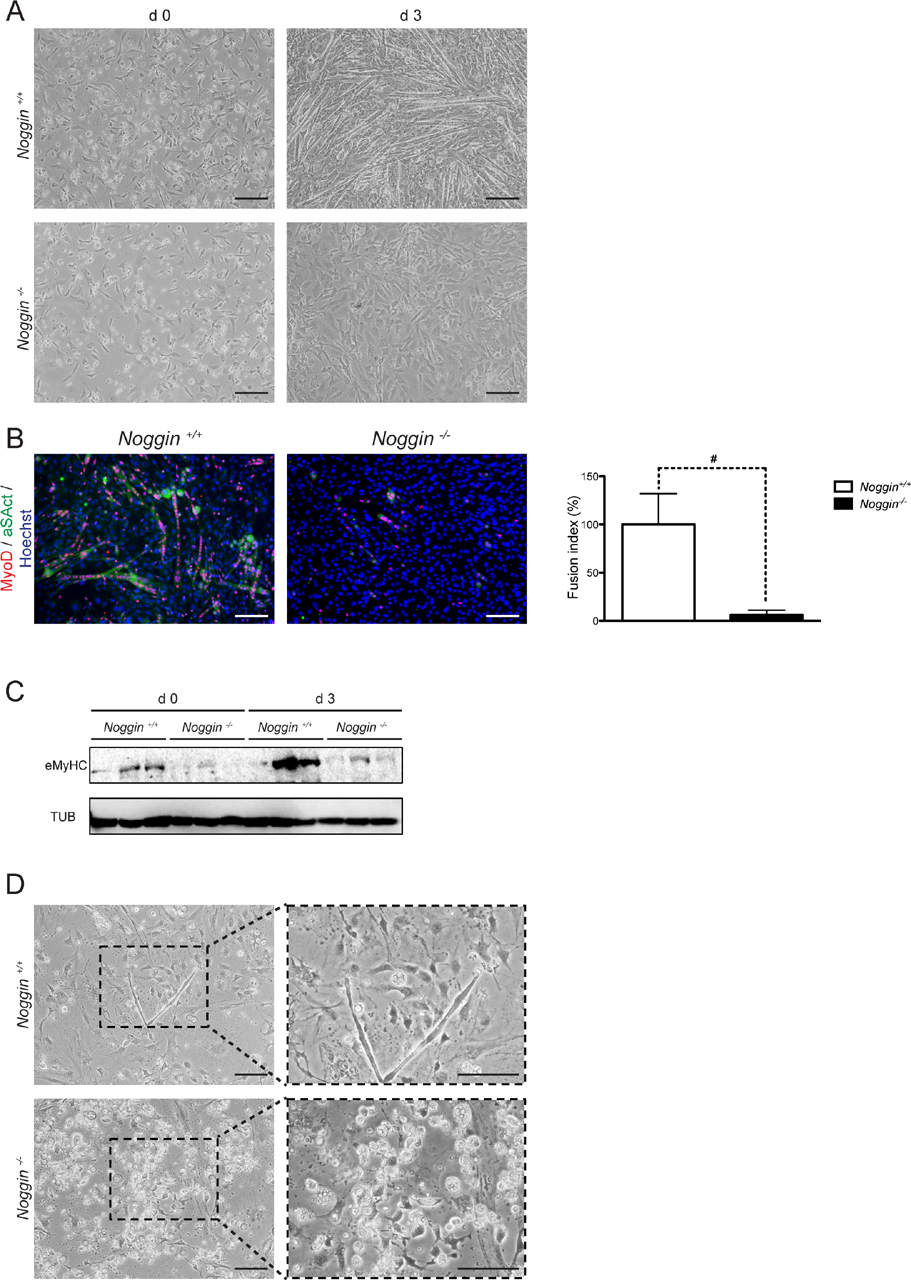

Supplement: Supplementary Figure S1 [file srep31949-s1.jpg]
